# Supplementary material for: Investigating the Cellular Uptake of Model Nanoplastics by Single-Cell ICP-MS
Source: Nanomaterials (Basel). 2023 Feb 1;13(3):594. doi: 10.3390/nano13030594 (PMC9920308; doi:10.3390/nano13030594)
Supplement: Supplementary file 1 [file nanomaterials-13-00594-s001.zip › nanomaterials-2127064-supplementary.pdf]

Investigating the Cellular Uptake of Model Nanoplastics by Single Cell ICP-MS

Domenico Cassano [1], Alessia Bogni [1], Rita La Spina [1], Douglas Gilliland [1], and Jessica Ponti [1,*]

[1] European Commission, Joint Research Centre (JRC), Ispra, Italy
* Correspondence: jessica.ponti@ec.europa.eu

Supporting Information

Figure S1

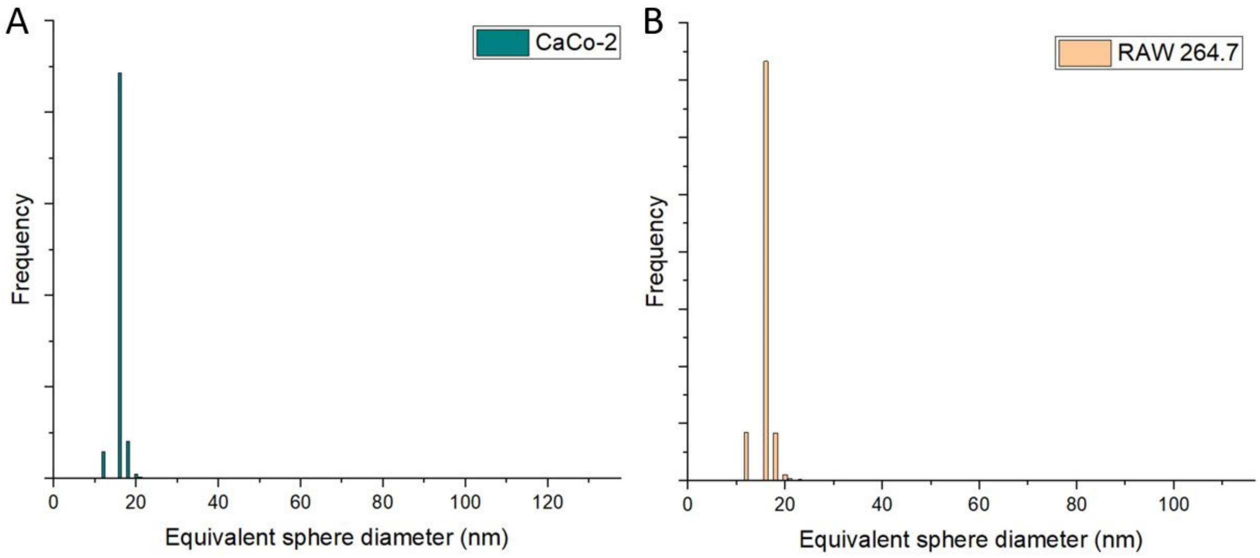

***Figure S1**. Equivalent sphere diameter of control CaCo-2 (A) and RAW 264.7 (B) cells. The distributions are almost identical and are likely arising from background noise of scICP-MS instrument.*

Figure S2

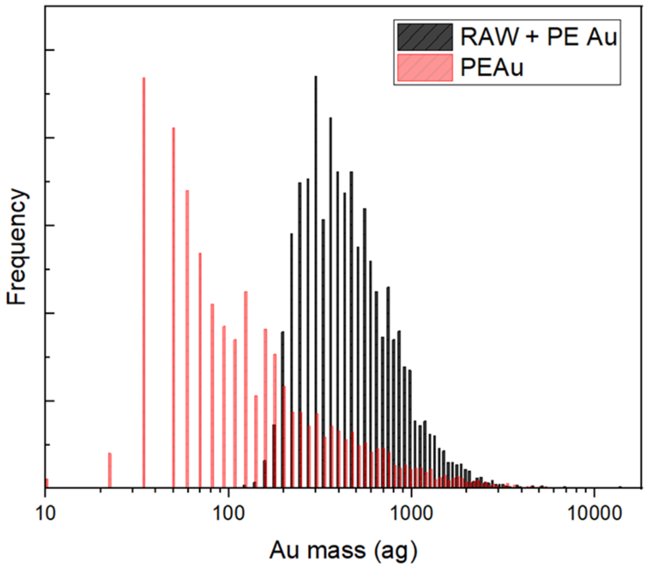

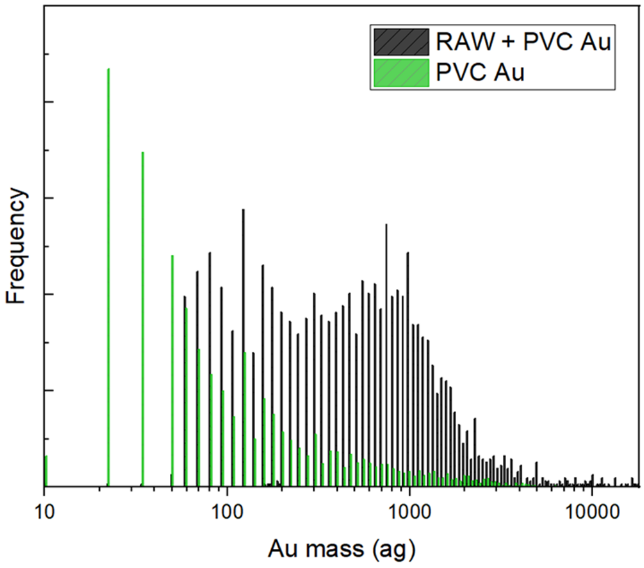

Figure S2. *(left) Au mass distribution of PE Au (red) and RAW 264.7 cells incubated with PE Au for 48 hours (black) at the concentration of 1 µg mL*$^{-1}$*. (right) Au mass distribution of PVC Au (green) and RAW 264.7 cells incubated with PVC Au for 48 hours (black) at the concentration of 1 µg mL*$^{-1}$*.*

Figure S3

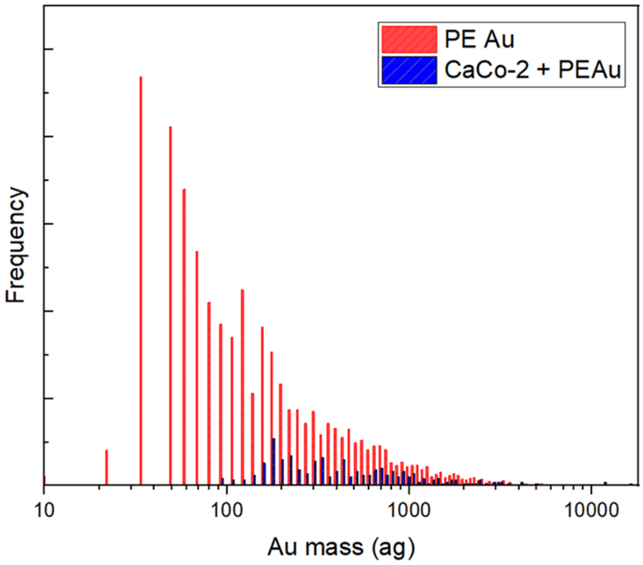

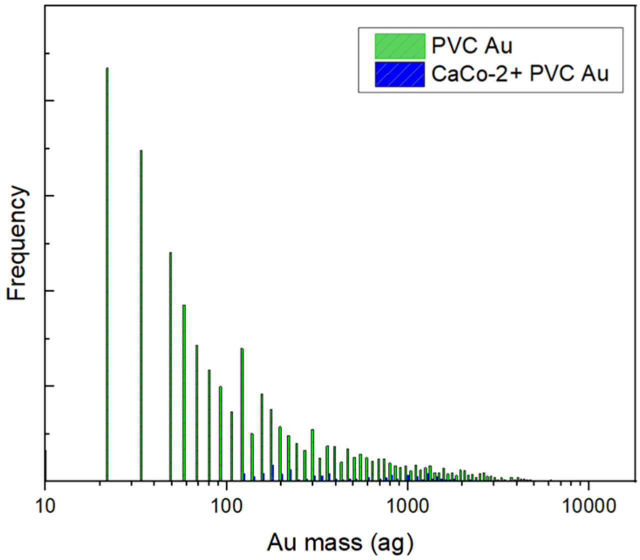

Figure S3. *(left) Au mass (actograms) distribution of PE Au (red) and CaCo-2 cells incubated with PE Au for 48 hours (blue) at the concentration of 1 µg mL*$^{-1}$*. (right) Au mass (actograms) distribution of PVC Au (green) and CaCo-2 cells incubated with PVC Au for 48 hours (blue) at the concentration of 1 µg mL*$^{-1}$*.*

Figure S4

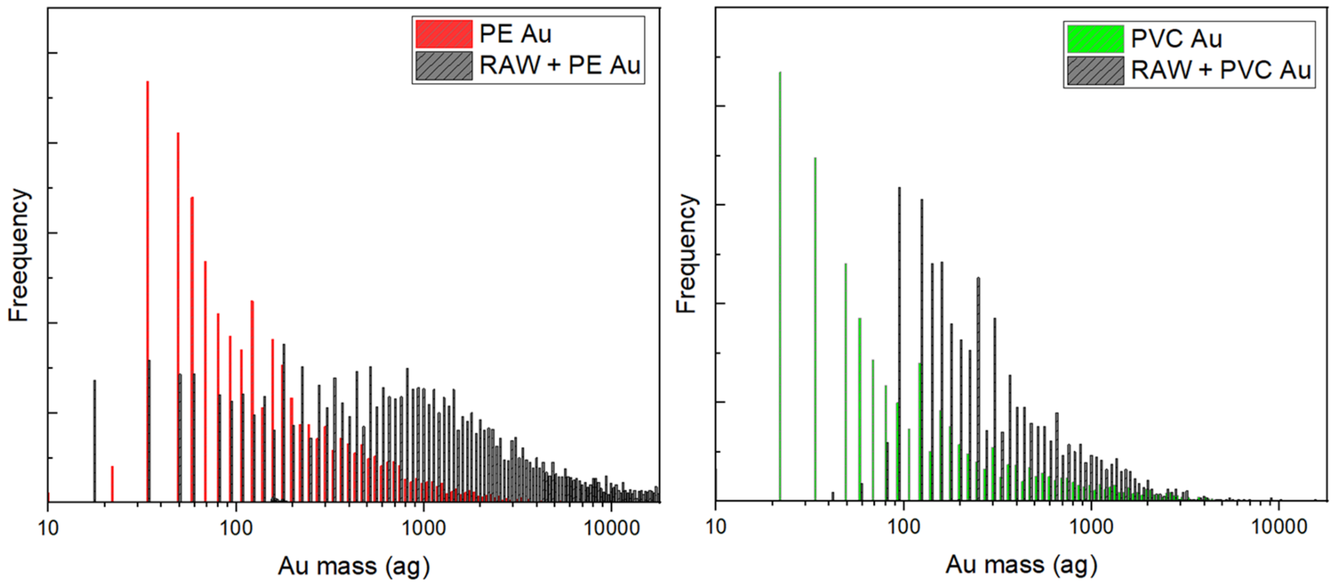

Figure S4. *(left) Au mass (actograms) distribution of PE Au (red) and RAW 264.7 cells incubated with PE Au for 48 hours (black) at the concentration of 100 µg mL^{-1}. (right) Au mass (actograms) distribution of PVC Au (green) and RAW 264.7 cells incubated with PVC Au for 48 hours (black) at the concentration of 100 µg mL^{-1}.*

Figure S5

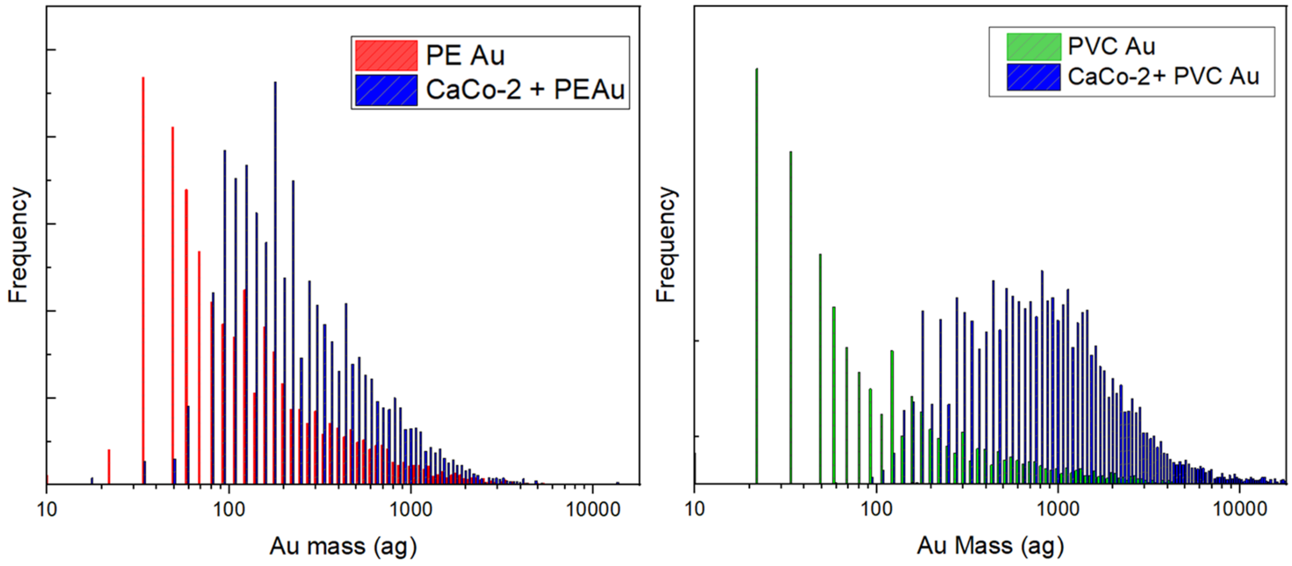

Figure S5. *(left) Au mass (actograms) distribution of PE Au (red) and CaCo-2 cells incubated with PE Au for 48 hours (blue) at the concentration of 100 µg mL^{-1}. (right) Au mass (actograms) distribution of PVC Au (green) and CaCo-2 cells incubated with PVC Au (blue) for 48 hours at the concentration of 100 µg mL^{-1}.*
